# Microfluidic-Assisted Formulation of ε-Polycaprolactone Nanoparticles and Evaluation of Their Properties and In Vitro Cell Uptake

**DOI:** 10.3390/polym15224375

**Published:** 2023-11-10

**Authors:** Ewa Rybak, Piotr Kowalczyk, Sylwia Czarnocka-Śniadała, Michał Wojasiński, Jakub Trzciński, Tomasz Ciach

**Affiliations:** 1 Faculty of Chemical and Process Engineering, Warsaw University of Technology, Waryńskiego 1, 00-645 Warsaw, Poland; piotr.kowalczyk.dokt@pw.edu.pl (P.K.); michal.wojasinski@pw.edu.pl (M.W.); jakub.trzcinski@pw.edu.pl (J.T.); tomasz.ciach@pw.edu.pl (T.C.); 2 Nanosanguis S.A., Rakowiecka 36, 02-532 Warsaw, Poland; s.czarnocka@nanogroup.eu; 3 Centre for Advanced Materials and Technologies CEZAMAT, Warsaw University of Technology, Poleczki 19, 02-822 Warsaw, Poland

**Keywords:** polymeric nanoparticles, nanoprecipitation, microfluidic, cytotoxicity

## Abstract

The nanoprecipitation method was used to formulate ε-polycaprolactone (PCL) into fluorescent nanoparticles. Two methods of mixing the phases were evaluated: introducing the organic phase into the aqueous phase dropwise and via a specially designed microfluidic device. As a result of the nanoprecipitation process, fluorescein-loaded nanoparticles (NPs) with a mean diameter of 127 ± 3 nm and polydispersity index (PDI) of 0.180 ± 0.009 were obtained. The profiles of dye release were determined in vitro using dialysis membrane tubing, and the results showed a controlled release of the dye from NPs. In addition, the cytotoxicity of the NPs was assessed using an MTT assay. The PCL NPs were shown to be safe and non-toxic to L929 and MG63 cells. The results of the present study have revealed that PCL NPs represent a promising system for developing new drug delivery systems.

## 1. Introduction

In recent years, the evolution of polymeric particles as drug delivery carriers has promoted the development of nano- and micro-medicine [1]. Due to their size, properly designed nanoparticles (NPs) can freely move throughout the body via the smallest capillaries, are easily administered by oral, pulmonary, vascular, and parenteral routes, and do not require surgical resection after complete administration. Due to the unique properties of some NPs, the biodistribution and pharmacokinetics of the encapsulated drug molecules can be altered, leading to improved efficacy, reduced side effects, and improved patient compliance [2]. NPs can be made to target desired cells and achieve controlled drug release; they could also bring about significant changes in medicine [3]. However, after first promising results and plans, we now understand that nanoparticle drug delivery technology still demands development and understanding to improve the final rate of delivery to the targeted cells [4].

Nanoparticles can be formulated from inorganic or organic–polymeric materials. Inorganic materials like gold, silica, or iron oxide are widely developed due to the vast number of synthetic protocols available. However, polymeric NPs have gained much attention as they can be precisely designed to achieve prominent biodegradability and biocompatibility [5]. The degradation in vivo of some desired polymers results in toxicologically safe side products that are further removed via the normal metabolic pathways or reused as nutrients. Biodegradable polymers are advantageous over other materials for use in drug delivery systems, such as nanoparticles. NPs can be customized into various shapes and sizes, with tailored pore morphologies, mechanical properties, and degradation kinetics. By selecting the appropriate polymer type, molecular weight, and copolymer blend ratio, the degradation/erosion rate of the nanoparticles can be controlled to achieve the desired style and rate of release of the encapsulated payload [6]. Biodegradable polymers can be generally classified as natural polymers, such as chitosan, hyaluronan, etc., and synthetic polymers that include poly-lactic-*co*-glycolic acid (PLGA) [7,8], polylactic acid (PLA) [9,10], or polycaprolactone (PCL) [11,12]. The aforementioned polymers have been widely used for drug encapsulation studies. PCL is a semi-crystalline polyester that is hydrophobic, biodegradable, and biocompatible. The glass transition temperature (T_g_) of −60 °C and low melting point (59–64 °C) of PCL allow for the easy fabrication of delivery systems at reasonably low temperatures [13]. Furthermore, PCL has excellent blend compatibility with other polymers, facilitating the tailoring of desired properties like degradation kinetics, hydrophilicity, and mucoadhesion [14,15]. PCL is an advantageous material for its high permeability to small drug molecules and, in comparison to polylactic and polyglycolic acid polymers, has an inessential tendency to generate an acidic environment during the degradation process, a problem that contributes to the generation of inflammatory reactions. The degradation of PCL is very slow compared to the other polyesters, making it more suitable for long-term delivery systems with the advantages of less frequent administrations, an increase in patient compliance, and the reduction of discomfort [16].

The methods of nanoparticle production have evolved in the last few decades. However, nanoparticles are primarily synthesized in a bench-top batch mode using basic experimental techniques and equipment, i.e., traditional beaker or stirred flask methods. These techniques involve various drawbacks, resulting in polydispersity in size distribution, particle structure, and particle properties [17]. Depending on the particular application and the formulation method, it is crucial to achieve the required characteristics of NPs [8]. There are various methods for NP formulation using biodegradable polymers, such as salting out, emulsification, solvent evaporation, monomer polymerization, or nanoprecipitation [18]. The nanoprecipitation method was first described by Fessi et al. [19], who reported a simple process for the fabrication of polymeric nanoparticles. It involves the precipitation of a dissolved material into nanoparticles after exposure to a polymer non-solvent (polymer precipitant) that is miscible with the solvent [20]. The rapid diffusion of the solvent into the non-solvent phase results in the decrease of interfacial tension between the two phases, which increases the surface area and leads to the formation of small droplets of organic solvent [19,21,22]. There are three stages of the formulation of nanoparticles: nucleation, growth by condensation, and growth by coagulation, which leads to the formation of polymer nanoparticles or aggregates [8,23]. The rate of each step determines the particle size, and the ratio of polymer concentration over the solubility of the polymer in the solvent and non-solvent mixture is the driving force of these phenomena. The critical factor for uniform particle formation is separating the nucleation and the growth stages [24,25]. Preferably, operating conditions should allow a high nucleation rate strongly dependent on supersaturation and a low growth rate [22]. Nanoprecipitation has been a widely recognized and established approach in the realm of nanoparticle formulation. Recently, it has been incorporated into the emerging concept of nanoarchitectonics [26,27,28]. However, even with the development of cutting-edge methods, in some cases, the procedure is still carried out in the same manner as it was two decades ago. Despite all the challenges, nanoprecipitation is a simple, fast, and reproducible method still widely used to prepare NPs [29,30,31,32,33,34].

Nevertheless, the successful adaptation of nanoparticle formulations still confronts numerous challenges, such as low production efficacy, high batch-to-batch variations, shorter residence time, and the substandard scale-up feasibility of the manufacturing process. Therefore, an approach that can formulate nanoparticles with desired physicochemical characteristics in a high-throughput and reproducible manner is strongly desirable [35]. 

The conventional methods typically used for polymeric NP preparation (i.e., dropwise method) usually exhibit a broad size distribution due to the lack of precise control over the mixing process. Alternatively, the microfluidics technique can be used for NP fabrication since it allows for rapid mixing and the precise control of different streams to achieve control over NP size and distribution [16,36,37,38,39].

Microfluidic technology has been used to formulate polymeric NPs with a high degree of control over particle size, shape, and composition. The main advantage of this technology is the conduction of physical or chemical processes in a small volume with mostly diffusive control of mass transport phenomena, which leads to a repeatable and controlled process as compared to large reactors and mixers. This approach offers a scalable manufacturing process for these materials, with potential applications in drug delivery, diagnostics, and sensing [25,39,40,41]. Microfluidic devices are used to provide accurate control over the size distribution, agitation, and shear forces [30]. Furthermore, microfluidic devices offer the advantage of reusability. Microfluidic devices typically consist of various components designed to control the fluid flow and enable efficient nanoparticle synthesis. The key components include fluidic inlets and reservoirs, microchannels, junctions, and mixers. A microfluidic device consists of channels typically ranging from 1 to 100 µm in hydraulic diameters [29]. These channels have walls with different geometries that create capillary action. This creates a narrow channel with a small cross-section that is well suited for the suspension of small particles. Narrow channels are more efficient at maintaining shear forces and particle positions during flow, which facilitates the formation of highly efficient dispersions. Additionally, channel dimensions less than 100 µm in diameter allow for high channel pressure without losing flow altogether. Polymeric nanoparticles can be formulated with microfluidic assistance to improve their properties [41].

Microfluidic techniques are widely used for the preparation of colloidal suspensions; however, they can also be used for other applications such as homogenization, heterogenization, and stirring homogeneous dispersions in inorganic materials. Since microfluidic chips can be designed to be very efficient mixers, the mixing rate between a solvent and non-solvent can shift the precipitation rate toward the high nucleation stage rather than the growth stage [22]. Moreover, using precise external equipment (e.g., syringe pumps or gear pumps), it is possible to reproduce synthesis protocol without variety in nanoparticle characteristics. Particle sizes can be precisely controlled using microfluidic assistance, which makes these formulas ideal for formulation purposes such as cosmetics or pharmaceutical formulation development [40,42]. 

This work compares a novel microfluidic strategy for fabricating polymeric NPs with the traditional nanoprecipitation method in a vessel. The microfluidic chip brings two co-flowing streams into contact in a specially designed flow-focusing device to enhance mixing. The main objectives of this study were to investigate the effect of operating parameters, system geometry, and polymer concentration on the final particle size distribution. Moreover, the cytotoxicity and dye-releasing behavior of synthesized polymeric NPs against L929 and MG63 cells were thoroughly examined to uncover their potential in cancer therapy.

## 2. Materials and Methods

### 2.1. Materials

ε-Polycaprolactone (PCL) with a weight average molar mass of 14,000 g/mol, Pluronic^®^F-127, PBS (phosphate-buffered saline) tablets, fluorescein, and 3-(4,5-dimethylthiazol-2-yl)-2,5-diphenyl tetrazolium bromide (MTT) were purchased from Sigma-Aldrich/Merck (Poznań, Poland), and dimethyl sulfoxide (DMSO) and tetrahydrofuran (THF) (HPLC grade, purity 99.9%) were obtained from Chempur (Piekary Śląskie, Poland). All the other reagents were of pharmaceutical grade and were used without further purification. The antisolvent phase was ultrapure water produced by reverse osmosis (Milli-Q^®^, Millipore, Burlington, MA, USA) with the addition of surfactant (Pluronic^®^F-127).

### 2.2. Microfluidic Device

**Figure 1:**
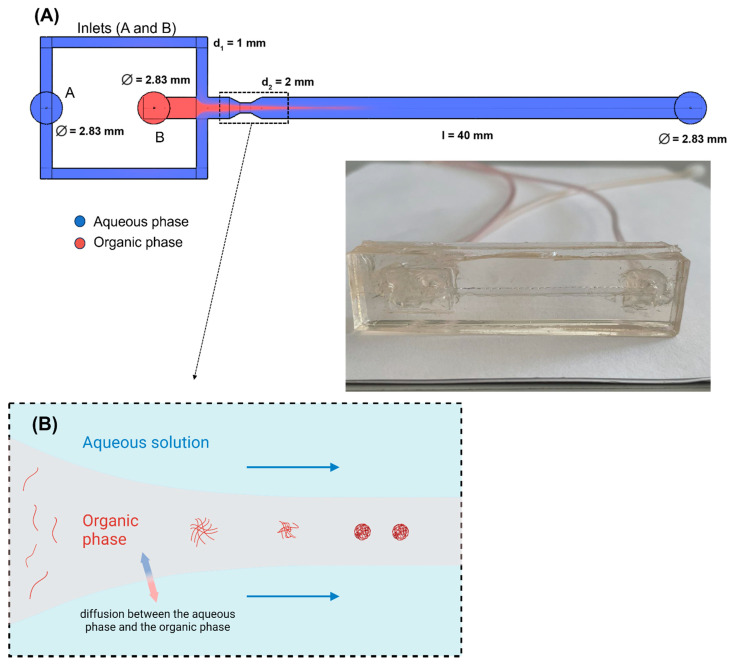
Flow-focusing microfluidic device for NP formulation. (**A**) Schematic of the microfluidic device. (**B**) Proposed mechanism of NP formulation. Created with BioRender.com, accessed on 17 October 2023.

The fluidic device was designed in Blender 3.0 software (Figure 1A). The inner channel geometry was saved as a .stl file and 3D-printed using a ZMorph VX printer (ZMorph, Wrocław, Poland). The printing material used was acetonitrile butadiene styrene (ABS) 1.75 mm filament (ZMorph, Wrocław, Poland). The printed model was then placed in a rectangular form, followed by polydimethylsiloxane resin—Sylgard 184 Silicone Elastomer (Dow Chemical, Midland, MI, USA). The mixed resin was degassed under the vacuum before application. After 15 min of curing at 90 °C, the 3Dprinted model was removed, and the hollow space left by the model was covered by the second flat piece of partially cured PDMS and a 1 kg weight to press the pieces together. The sandwiched device was further cured at 90 °C overnight. Silicone tubing with 3 mm diameter was installed in the PDMS chip inlets and outlet and sealed with a small amount of silicone glue. 

Figure 1 shows the design of our flow-focusing microfluidic device. It has two inlets and one outlet. The organic dispersed phase was introduced from the central channel (inlet B), and the continuous phase (aqueous solution) was introduced through inlet A. The organic and aqueous phases were pumped using KD Scientific and Ascor AP-14 syringe pumps, respectively. Syringes (Beckton Dickinson, Warsaw, Poland) were used for water and polymer/THF solutions. PTFE and silicon tubing (Cole Parmer, Vernon Hills, IL, USA) were used to connect the syringe and the microfluidic device. 

### 2.3. Methods

#### 2.3.1. Preparation of Polymeric Nanoparticles

##### Preparation of Solutions

To prepare the organic phase for blank emulsions, different amounts of PCL were dissolved in 10 mL of THF to form an organic phase with various polymer concentrations (0.1, 0.5, 1.0, 2.0, 5.0% *w*/*v*). For dye-loaded polymeric NPs, the organic phase was prepared by dissolving different amounts of PCL in 10 mL of THF until complete dissolution. Then, fluorescein (1/100 of used PCL amount *w*/*w*) was added with continuous stirring until complete dissolution. 

The antisolvent phase was an aqueous Pluronics^®^F-127 surfactant solution (0.6% *w*/*v*). The surfactant’s role was to stabilize the NPs of the dispersed phase after mixing both phases and prevent agglomeration, coalescence, and imperfect surface formation, as well as facilitate NP size focusing. 

##### Formulation of Nanoparticles

For the classic nanoprecipitation method in a beaker, the organic phase was added dropwise via the syringe pump (LEGATO 210; KD Scientific Inc., Holliston, MA, USA) at a constant rate (0.15 mL/min) to the aqueous phase (ultrapure water MilliQ, Millipore, Burlington, MA, USA) containing surfactant under magnetic stirring (1000 rpm) at room temperature. NPs were formed and the appearance of a milky colloidal suspension was observed. The obtained suspension was stirred magnetically for 10 min. Solvent evaporation was subsequently carried out under magnetic stirring for 72 h at room temperature. The obtained suspension was subjected to sonication and filtration on the syringe filter (0.45 μm).

For the NP formulation using the microfluidic device, the syringes containing organic and aqueous phases were placed in the syringe pumps and connected to the module. A concept of flow rate ratio (*R*) parameter was introduced, based on hydrodynamic-focusing research in the synthesis of polymeric nanoparticles [43].

$$R=\frac{V_{aq}}{V_{org}}[-]$$

Here:

*V_aq_*—flow rate of aqueous phase [mL/h]

*V_org_*—flow rate of organic phase [mL/h]

The precipitation process described above was carried out for six *R* values: 10, 20, 50, 100, 150, and 200 at room temperature. After examination of preliminary results, the flow rate ratio was set at 200. After completion of the process, solvent evaporation was carried out under magnetic stirring for 72 h at room temperature. Then, the obtained suspension was subjected to filtration on the syringe filter (0.45 μm). 

#### 2.3.2. Particle Size and Zeta Potential Analysis

The average particle sizes and polydispersity index (PDI) were measured using dynamic light scattering (DLS) using Malvern Zetasizer (Nano ZS, Malvern Instruments, Malvern, UK), equipped with a detector to measure the intensity of the scattered light at 173° to the incident beam. The zeta potential (Z-potential) of the aqueous dispersions was also determined using Zetasizer Nano ZS at 25 °C. All measurements were replicated at least three times and presented as mean values with standard deviations. 

#### 2.3.3. Scanning Electron Microscopy (SEM)

The NP morphologies and surface characteristics were investigated based on the images from a scanning electron microscope (SEM, SU8230, Hitachi, Chiyoda City, Tokyo, Japan). First, the NP suspensions were prepared. Following particle size and zeta potential analysis in Section 2.3.2, the samples were diluted 1000 times with ultrapure water and prepared for imaging. The NP diluted suspensions (10 µL) were placed on the surface of the silicon wafer, which was first glued with carbon tape to the aluminum stub. The suspensions on the surfaces of silicon wafers were left for evaporation overnight at ambient temperature (about 22 °C). Such preparation allowed the separate NPs to be imaged as single particles on the surface of the silicon wafer. Images were collected with a 10 kV accelerating voltage, at about 10 mm working distance, using an upper detector of scatted electrons (SE(U)).

#### 2.3.4. FTIR Analysis 

Analysis of the chemical interactions of the freeze-dried blank NPs and the loaded NPs was performed using Fourier Transform Infrared Spectroscopy (FTIR) using Nicolet 6700 FTIR (ThermoFisher Scientific^®^, Waltham, MA, USA). The samples were prepared by mixing the sample fine powder obtained after the lyophilization with IR-grade KBr and subsequent pressing. The scanning range was 4000–500 cm^−1^. 

#### 2.3.5. In Vitro Release Studies

In vitro release of fluorescein from nanoparticles was investigated for three selected formulations from each method (dropwise and microfluidic methods). NP fractions of 1% PCL were chosen as they were characterized by the smallest mean diameter and PDI value. The release of fluorescein from PCL NPs was investigated using a dialysis membrane tubing—regenerated cellulose with a molecular cut-off of 12,000–14,000 Dalton (Spectra/Por Membranes, Spectrum Laboratories, Inc., Rancho Dominguez, CA, USA). Membrane tubes were filled with 3 mL of chosen formulations, sealed with dialysis clips, and then placed in a glass beaker containing 200 mL of PBS and dimethyl sulfoxide (DMSO) solution (4:1 *v*/*v*). Experiments were carried out at room temperature for 24 h, and sink conditions were maintained during the analysis. At 0 h, 1 h, 2 h, 3 h, 4 h, and 24 h, 1 mL of the receptor medium was withdrawn, replaced with the same volume of a fresh solution of PBS and DMSO, and then immediately analyzed using a UV-Vis spectrophotometer at 490 nm (BMG, Labtechnologies, Offenberg, Germany). Each measurement was performed in triplicates. Fluorescein solutions ranging from 0.001 to 0.1 mg/mL were prepared to construct a calibration curve that was used to quantify the payload released from the NPs, according to the following equation:

y=102.17x+0.1401

where *x* represents the fluorescein concentration (mg/mL) and *y* is the UV/Vis absorbance (nm). The *R*^2^ value was 0.9912. No interference was observed at fluorescein λ_max_ of 490 nm from other components of the formulation. The dye encapsulation efficiency (*EE_%_*) was calculated as the ratio between the unbound dye and the total dye concentration in nanosuspension, according to the following equation:

$$EE_{\%}=\frac{m_{dye\;total}-m_{dye\;unbound}}{m_{dye\;total}}*100$$

The drug loading (*DL_%_*) was calculated as the mass fraction of dye in the NPs, according to the following equation:

$$DL_{\%}=\frac{m_{dye\;total}-m_{dye\;unbound}}{m_{NPs}}*100\;$$

#### 2.3.6. Cellular Uptake and Cytotoxicity Assay

To determine the controlled release of the payload from fluorescent NPs, the NPs were incubated with mammalian cells. The fluorescein-loaded NPs were added into the culture of the L929 mouse fibroblast cell line and MG63 human osteosarcoma cells with fibroblast morphology. Fibroblasts are known to be pivotal in contributing to the progression of several malignancies, including endometrial cancer, and therefore represent a possible target for nanoparticle-based therapeutic approaches.

##### Cytotoxicity Assay

MG63 and L929 cell viability was determined using the standard 3-(4,5-dimethylthiazol-2-yl)-2,5-diphenyl tetrazolium bromide (MTT) assay 24 h after exposure to NPs. Cells suspended in culture medium at the density of 1 × 10^5^/mL (100 µL per well) were seeded onto 96-well plates and cultured for 24 h at 37 °C and 5% CO_2_ to adhere. Next, NPs at a series of dilutions—0.01, 0.1, 1.0, and 10 mg/mL—were added (12 wells per variant). Untreated cells served as a positive control of viability. Cells were incubated under standard conditions for 24 h. Then, the NP-containing medium was removed, cells were rinsed three times with PBS, and MTT solution in medium (final MTT concentration 1 mg/mL) was added and incubated (3 h, 37 °C). The MTT solution was removed without disturbing cells, 100 µL/well DMSO was added, the plates were shaken gently (5 min) to dissolve formazan crystals, and the absorbance was read on a microplate reader at 570 nm and 650 nm. 

##### Confocal Microscopy

MG63 and L929 cell lines suspended in culture medium at the density 1 × 10^5^ cell/mL were seeded onto a 24-well plate with a glass plate in each well and cultured for 24 h at 37 °C and 5% CO_2_ to adhere. Next, cells were treated with NPs at the concentrations of 1 mg/mL and 0.1 mg/mL (1 mL per well) for 1 h, 2 h, and 24 h under standard conditions. After stimulation, cells were fixed with 4% paraformaldehyde for 15 min, and after washing with PBS, cells were exposed to DAPI (4′,6-diamidino-2-phenylindole) to stain cell DNA. The cells were visualized with a confocal microscope (Zeiss) at the appropriate wavelengths for fluorescein (excitation 470 nm, emission 519 nm) and for DAPI (358 nm excitation, 461 nm emission) at magnification of 20×. Four independent repetitions for each experimental variant were performed. 

## 3. Results and Discussion 

**Figure 2:**
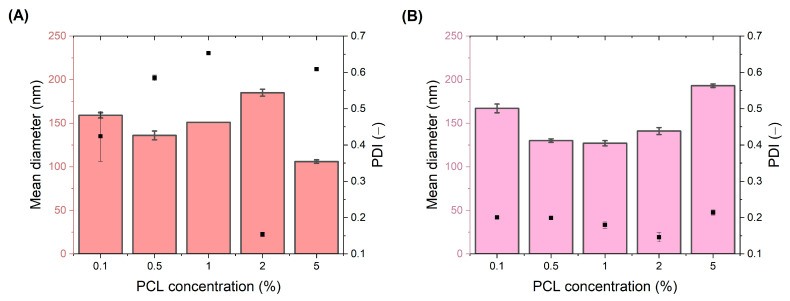
Comparison of the microfluidic device with the dropwise nanoprecipitation method—mean diameter of dye-loaded NPs and PDI (black dot). Dropwise DNPs (**A**), microfluidic device MNPs (**B**).

The particle sizes of NPs formulated dropwise (DNPs) were found to be from 106 to 185 nm, with a polydispersity index (PDI) of 0.154–0.653 (Figure 2A) for dye-loaded DNPs and 0.090–0.252 for blank DNPs (see Supporting Information). The values of PDI were higher for DNPs compared to the NPs formulated with the use of the microfluidic device (Figure 2B). The PDI values of blank microfluidic NPs (MNPs) were between 0.060 and 0.150 (Table S1. Supporting Information), while the PDI of dye-loaded MNPs was between 0.146 and 0.214, with the NPs’ mean diameter from 127 to 193 nm, which was higher than that of the DNPs. The minimum dye-loaded particle size of 106 ± 2 was achieved for DNPs while the smallest polydispersity index (0.146 ± 0.013) was achieved for MNPs. The particles formulated with the microfluidic device were more uniform in size even when higher polymer concentrations were used. Although the size distribution of MNPs was slightly higher than that of DNPs for the analyzed polymer concentration range, the sizes of MNPs were smaller for PCL concentrations of 0.5%, 1%, and 2%. This suggests that the microfluidics method was able to produce NPs with the desired size and PDI over a wider range of polymer concentrations than the dropwise method.

When the polymer content in the organic phase is increased, the viscosity rises due to higher mass transfer resistance, resulting in larger NP formation. This observation is consistent for 0.5, 1, and 2 polymer mass percentages in the dropwise addition method. The only discrepancy is present in the case of the 0.1 and the 5 mass percentages of the polymer (Figure 2A), where contradictory results were observed. This may be justified by the fact that at the highest polymer concentration, more nuclei were obtained to formulate NPs with small sizes. The PDI value (>0.2) supports uneven nanoparticle growth phenomena, which may be associated, as well, with aggregation tendency. 

Formulated NPs have a negative surface charge, which can be a result of the carbonyl group of the PCL polymer present at the surface of the nanoparticle structure [44]. The zeta potential values ranged from −14.6 to −21.0 mV across all formulations, and the method of formulation and variation in polymer concentration did not impact these values. As a result, it can be inferred that the NPs will remain physically stable [45]. 

Since strict control over a nanoparticle’s physical properties is pivotal for nanomedicine usage, the NPs with the smallest mean diameter and PDI were chosen for further research (1% PCL).

### 3.1. SEM Analysis

**Figure 3:**
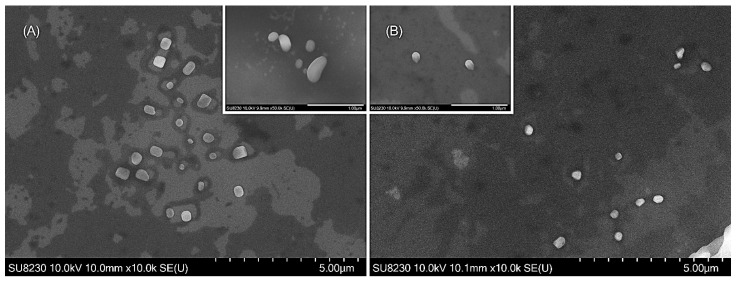
Scanning electron microscopy (SEM) images of (**A**) DNPs and (**B**) MNPs. Inserts show the smallest particles within the investigated samples. Insert scale bars represent 1 μm.

Fluorescein-loaded NPs’ morphology was visualized using SEM and prepared NPs were assessed in terms of size, shape, and smoothness. Representative images of 1% PCL DNPs and MNPs are presented in Figure 3. The prepared NPs have smooth surfaces. Notably, the MNPs (Figure 3B) have a smaller mean diameter and a narrower size distribution than the DNPs (Figure 3A), which is coherent with values obtained using the DLS technique. DNPs have elongated shapes while MNPs are almost spherical as a result of a controlled formation process. The microfluidic device has a short mixing time and residence time, which prevents the excessive growth of particles. Additionally, the soluble molecules move uniformly in all directions towards the nucleus surface, resulting in spherical particles that minimize surface energy and maintain stability [46]. On the other hand, in the dropwise method, the nucleation and growth mechanisms of NPs cannot be separated due to the lack control of precise control over mixing conditions, leading to excessive particle growth that affects particle size and shape [29,47,48]. The morphology of the NPs appears to be slightly distorted, which could be attributed to the facile dissolution of surfactant on the NPs’ outer surface. This phenomenon leads to the exposure of PCL and may also result in the gradual dissolution of the exposed PCL over time [49]. 

### 3.2. FTIR Analysis

**Figure 4:**
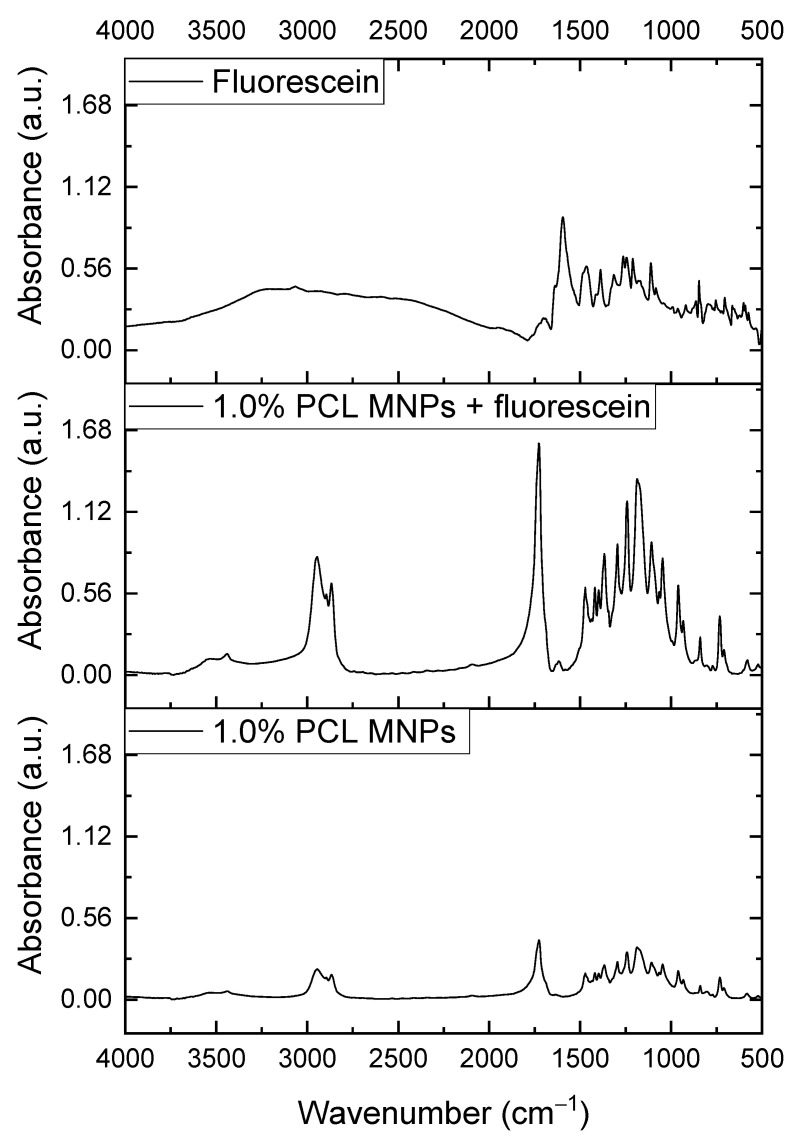
FTIR spectrum of pure fluorescein (**top**) and 1.0% PCL MNPs with (**middle**) and without (**bottom**) fluorescein.

To confirm nanoparticle chemical composition, FTIR analysis was carried out on pure fluorescein, blank MNPs, and MNPs loaded with fluorescein (Figure 4). All compounds presented their characteristic bands. The MNPs showed a band with asymmetric and symmetric stretching of C-H_2_ at 2940 cm^−1^ and 2869 cm^−1^, respectively. Moreover, a peak corresponding to carbonyl stretching at 1722 cm^−1^ was observed as well. The band at 2867 cm^−1^ was related to the stretching vibration of the C–H bond from Pluronic^®^F-127; at 1187 cm^−1^, a band of the C–O bond stretching appeared [50]. For fluorescein, the C=C stretching was observed within the range of 1643–1465 cm^−1^ [51]. Increasing PCL concentration in NP formation process does not change the shape of the spectrum; it only decreases the peak intensity (Figure S1, Supporting Information). This report supported the successful encapsulation of fluorescein into the formulated nanoparticles.

### 3.3. In Vitro Release Studies

**Figure 5:**
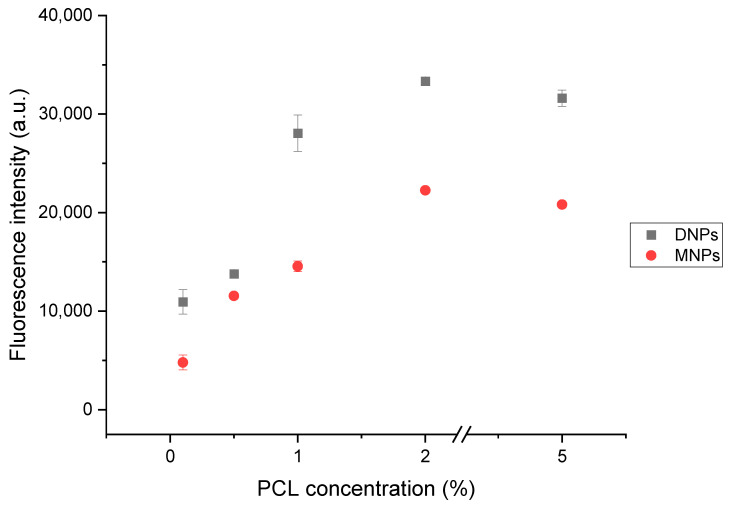
The relation between fluorescence intensity and increasing concentration of fluorescein-loaded DNPs and MNPs (1% PCL = 10 mg/mL).

The fluorescent labeling of the NPs was performed to study their biological localization. We prepared different formulations of NPs with various amounts of polymer used and proportional amounts of dye used. Based on the data presented in Figure 5, it can be observed that the fluorescence intensity of fluorescein is noticeably augmented as the concentration of fluorescein-loaded DNPs and MNPs increases. Notably, the fluorescence intensity values for DNPs are higher compared to those for MNPs.

The in vitro dye release from the fluorescein-loaded DNPs and MNPs was conducted in PBS (pH 7.4) containing DMSO (4:1 *v*/*v*). DMSO was used to enhance the solubilization of the hydrophobic dye. Because of the very low aqueous solubility of fluorescein, the addition of this solubility-enhancing component was necessitated to ensure the sink conditions and to achieve detectable UV/VIS concentrations during the release studies [52]. The dye release mechanisms are important in these formulations because of the proposed applications in sustained drug delivery. 

**Figure 6:**
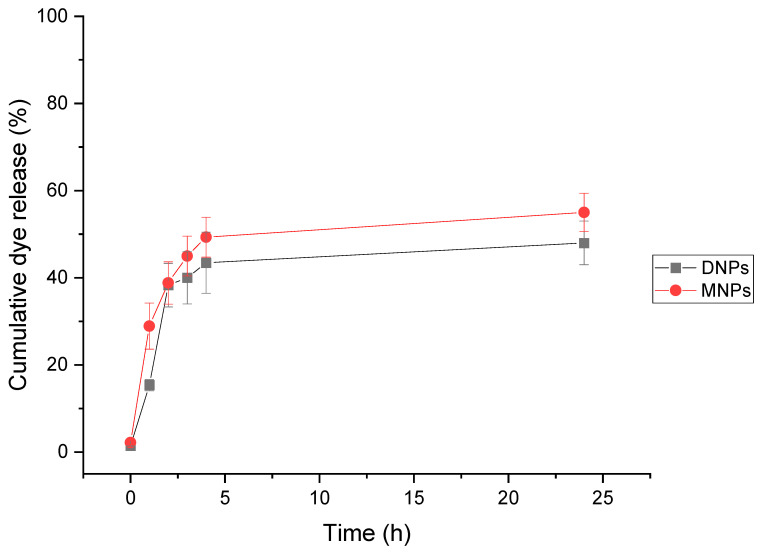
The cumulative release profiles of fluorescein from 1% PCL DNPs and MNPs of three independent measurements. The samples were measured at 0 h, 1 h, 2 h, 3 h, 4 h, and 24 h. Data are presented as means ± SDs.

Figure 6 illustrates that the dye-loaded PCL NPs manifested a standard biphasic dye release pattern from the nanoparticle matrix. The dye release profile displayed an initial burst release of 44% for DNPs and 49% for MNPs within the first 4 h, followed by a sustained dye release from the polymer matrix for 24 h. The initial phase of dye release is mainly attributed to the desorption or diffusion of the dye located on the large surface area of the nanoparticles or loosely bound to the polymer matrix. The remaining unreleased dye is thought to be tightly associated with PCL molecules and/or well trapped in the nanoparticle matrix and originates primarily from the diffusion or erosion of the matrix under sink conditions. If the diffusion of the dye is faster than matrix erosion, the release mechanism is largely controlled by the diffusion process [29,53,54,55]. 

In this study, both groups had relatively small mean diameters—DNPs at 151 nm and MNPs at 127 nm. During the experiment, it was noticed that DNPs exhibited a lower cumulative dye release of 48% compared to MNPs, which showed a relatively higher cumulative release of 55%. This difference could be explained by the fact that smaller nanoparticles possess a larger surface area, which results in a higher concentration of dye molecules at the surface of the NPs, ultimately leading to a faster dye release [53,54,56]. This observation has significant implications in the field of drug delivery as it provides a deeper understanding of the impact of particle size on drug release kinetics. 

**Figure 7:**
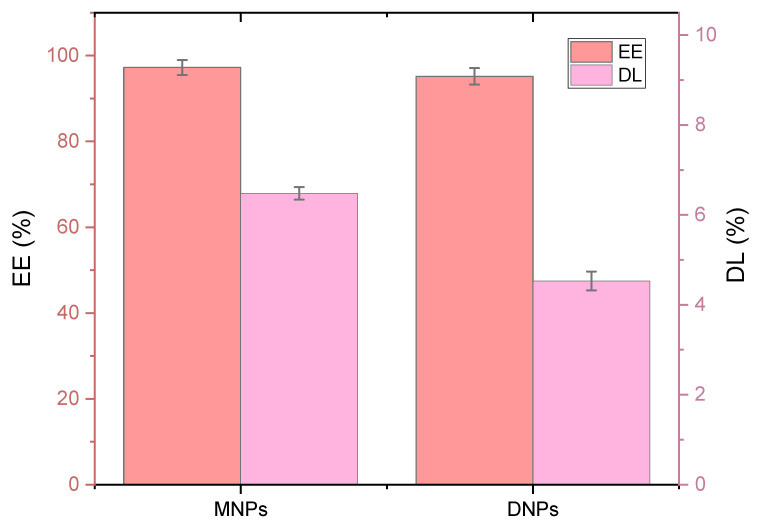
Drug loading and encapsulation efficiency [%] of fluorescein-loaded 1% PCL MNPs and DNPs.

The *EE_%_* and *DL_%_* of the fluorescein DNPs and MNPs are presented in Figure 7. The percentages of fluorescein loaded in the DNPs and MNPs were 4.53% and 6.48%, respectively. The encapsulation of the DNPs was 95.17%, and that of the MNPs was 97.22% (*n* = 3). It could be seen that both parameters reached higher values for MNPs. 

### 3.4. In Vitro Cytotoxicity and Cellular Uptake Studies

**Figure 8:**
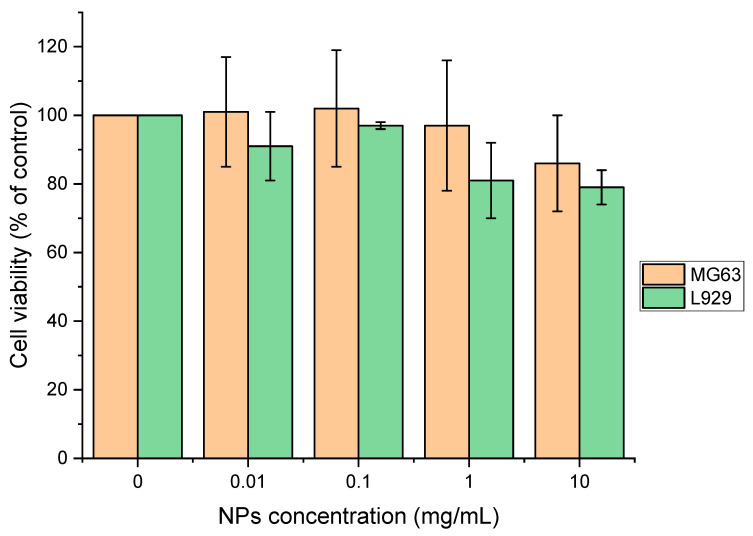
Cytotoxicity assessment of 1.0% PCL MNPs in two different cell lines, osteosarcoma cells MG63, and mouse fibroblast cell line L929. Cell viability (%) was calculated as (B)/A * 100), where A and B are the absorbances of control and treated cells, respectively. Values represent means ± SDs (*n* = 6).

MNPs are the preferred choice when conducting cell experiments due to their beneficial characteristics. Compared to DNPs, they have a smaller mean diameter and PDI value, along with higher *EE_%_* and *DL_%_* values. Additionally, MNPs demonstrate a controlled cumulative release profile, making them an ideal choice for such experiments. The formulated nanoparticles were examined for their potential cytotoxicity. Different concentrations of PCL NPs were added into the cell cultures (MG63 cell line and L929 cell line) and tested for their toxicity under 24 h of incubation. The results are presented in Figure 8. For MG63 cells, dosages of 0–10 mg/mL nanoparticles were examined for cytotoxicity, and in these examinations, PCL NPs showed no noticeable toxicity. This non-toxic trend can be observed until 10 mg/mL (cell viability then dropped to 86 ± 14%). In comparison, L929 cells showed elevated sensitivity as the dosage of NPs over 0.1 mg/mL started to lower the cell viability to 81 ± 11%, and to 79 ± 5% for the dosage of 10 mg/mL of NPs. Therefore, there are slightly different toxicity profiles for each cell line from the same nanoparticles. 

**Figure 9:**
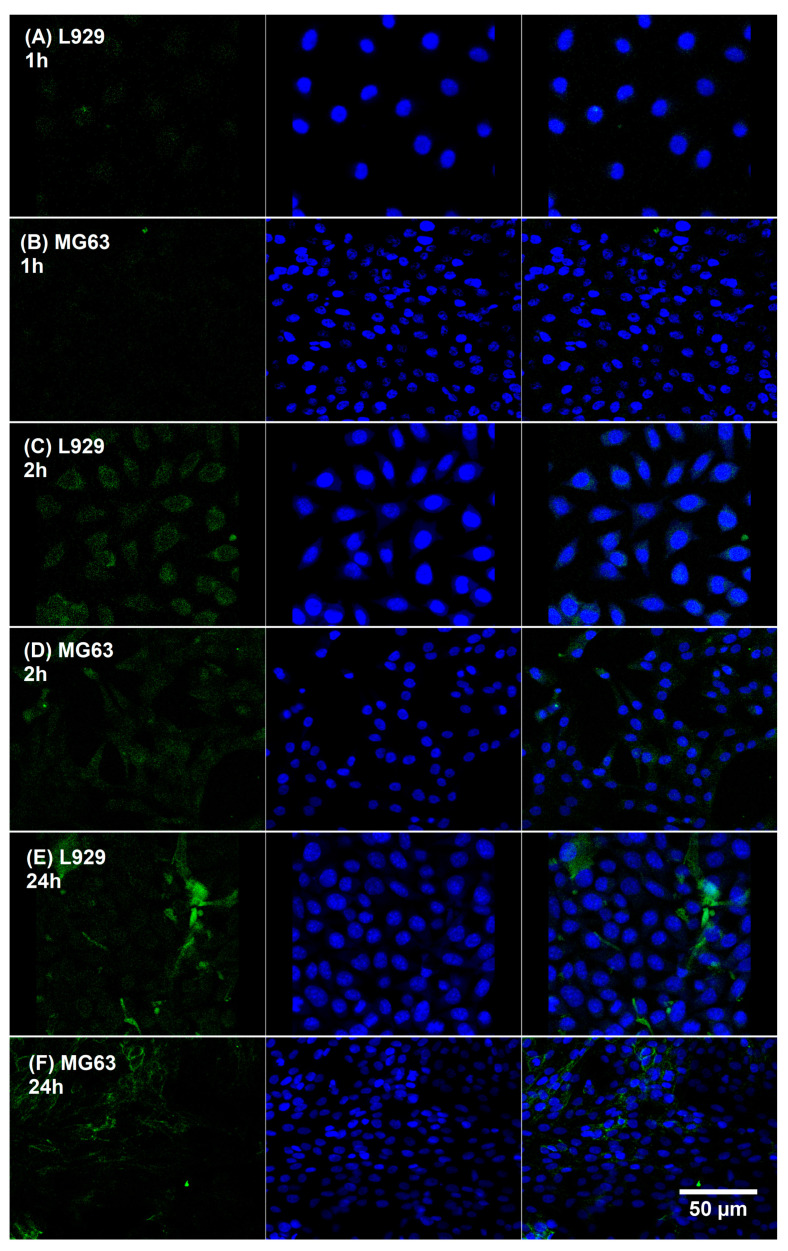
Confocal microscopy images of L929 and MG63 cells after 1, 2, and 24 h incubation at 37 °C with PCL MNPs with 120 nm in diameter (NPs concentration 1 mg/mL). Fluorescein (**left**), DAPI (**middle**), combined (**right**). Magnification: 20×; 1.0% PCL.

Finally, we performed confocal microscopy to study the effect of dye-loaded NPs composed of PCL in L929 and MG63 cells. Importantly, our results (Figure 9) supported data obtained via MTT assay, as shown in Figure 8. The dye-loaded NPs seemed to be effective in the delivery of payload in only an hour to both normal and cancerous cells. Figure 9 presents images of cells treated with NPs at the concentration of 1 mg/mL. For 0.1 mg/mL, the results were similar.

To evaluate the penetration of the NPs into the cells and the targeting effects of the loaded NPs, cellular uptakes of the MNPs were performed using L929 and MG63 cell lines. The lipophilic dye, fluorescein, was chosen in this study because the water solubility of fluorescein is poor and it could be easily encapsulated into the hydrophobic cores of the NPs [57]. The internalization of fluorescein-loaded NPs incubated for 1, 2, and 24 h was visualized using confocal laser scanning microscopy (CLSM). To observe the cellular distributions of NPs, we visualized green fluorescence from fluorescein and blue fluorescence from DAPI nuclei labeling. The fact that nanoparticle uptake by L929 and MG63 cells was significantly higher at 24 h compared to 1 or 2 h of incubation highlights the time-dependent accumulation of these nanoparticles within the cells. It was demonstrated that particle size determines both the mechanism and rate of intracellular uptake as well as the ability of a particle to permeate through tissue [58,59]. Indeed, the size of a particle can affect the efficiency and pathway of its cellular uptake by influencing its adhesion and interaction with cells [60]. A qualitative approach to cellular fluorescence revealed a gradual increase over 24 h, with evidence of a significant increase in payload release over time (Figure 9). Moreover, after 24 h, we could observe that the dye was cumulating in regions around the nucleus (Figure 9E,F). These results indicate that NPs loaded with a high fluorescein concentration release their cargo intracellularly. We suggest that lysosomal acid hydrolases may facilitate release in our experiments after the initial distribution of NPs in early endosomes and lysosomes due to the presence of PCL, a polyester highly sensitive to hydrolases [61]. The hydrolysis of the polyester leads to the destabilization of the vesicle structure, possibly leading to the accelerated release of fluorescein at physiological temperature (~37 °C) compared to the storage temperature (room temperature) of the NP formulation. Further high-resolution imaging studies will allow the better description of this process. 

Controlling ordered polymeric nanoparticles’ pore size, morphology, and particle size is significant for biomedical applications. Nano-sized particles (<200 nm) are better carriers than bulky polymeric materials with sizes greater than 1 µm as they exhibit rapid mass transfer, efficient adhesion to substrates, and good suspension in solution. Nanoparticles bigger than human albumin and smaller than approximately 200 nm have a better chance of staying in the circulatory system for a longer period [62]. Therefore, in this paper, polymeric nanoparticles with diameters below 200 nm are suggested to be well sized for cellular uptake. Size effects on cellular uptake are expected to result in size-dependent biochemical responses [58,63]. However, the particular responses of downstream cells require further investigation. 

## 4. Discussion and Conclusions 

Advances in nanomedicine require developing more robust and controllable procedures for manufacturing nanoparticles. Conventional methods rely heavily on bulk mixtures with poor batch-to-batch reproducibility, making it difficult to rapidly screen and optimize the properties of nanomaterials. There are several limitations associated with conventional methods of producing NPs. One such limitation is the need for additional chemical and physical processes such as freeze–thaw, high-pressure homogenization, and extrusion [64]. These processes not only require extra resources but also affect the quality and efficacy of the final product. Another drawback is insufficient macro-mixing, which can result in the uneven distribution of active ingredients. Additionally, there is a risk of potential contamination during the production process. These limitations pose a significant challenge in translating NPs from the laboratory to clinical use. Consequently, exploring and developing methodologies that can effectively tackle the obstacles and produce nanomaterials with utmost accuracy and precision is imperative. This critical aspect demands considerable attention and innovation from researchers and industry practitioners alike. The utilization of microfluidic devices presents a viable solution to the limitations of bulk mixing and top–down approaches given these devices’ microscale dimensions and ability to precisely control flow parameters, particle size tunability, and reproducibility. In bottom-up approaches, bulk mixing is influenced by various factors, such as temperature, precursor concentration, time, and pH. However, microfluidic NPs are further impacted by total flow rate, flow rate ratio, and residence time due to the continuous flow operation of microfluidics. These additional factors impact the physicochemical properties of NPs and the aforementioned parameters [65]. The miniature size of microfluidic tools also offers low power consumption, precise laminar flow due to small Reynolds numbers where viscous forces dominate, and improved mass and heat transfer due to large surface area. Aside from advantages, the main disadvantage is that these tools rely on novelty and not thoroughly investigated ongoing processes. Hence, microscale reactions become more complex and dependent on the process parameters than macroscale reactions. Microfluidic emulsion droplets are produced at tens to hundreds of microliters per minute, so emulsification is a lengthy process with low throughput per hour. Some groups have tried to overcome this situation by parallelizing the droplet generators [66], which can significantly increase a plant’s cost. 

In this study, size-tunable PCL NPs were prepared using the conventional nanoprecipitation method and a specially designed microfluidic device. For the microfluidic method, we tested six different *R* values, and after a thorough data analysis, the flow rate ratio was set at 200. The increased amount of water reduces the tendency of the particles to agglomerate because the particles collide and stick together less frequently. The particle buildup is most likely to occur near the orifice, with high localized particle concentrations. In addition, higher flow rate ratios ensure faster mixing in microfluidic systems [67]. With a faster mixing process, the critical supersaturation required for nucleation is reached more quickly, resulting in the formation of more nuclei whose growth is limited by the amount of polymer available in the liquid phase. Therefore, the higher the number of nuclei is, the smaller the nanoparticle size is. Laoini et al. also observed smaller particle sizes at higher *R* ratios in the production of liposomes and polymeric micelles in membrane contractors [68,69]. Jahn et al. observed these in the formation of liposomes in a planar flow-focusing microfluidic mixer [70], and Othman et al. did the same in the formulation of polymeric NPs using glass capillary micromixers [16]. A high flow rate allowed small-sized NPs to be produced with a low polydispersity index. In addition, a microfluidic device can prepare PCL-based dye-loaded NPs with a wide size range (127–193 nm) and lower PDI (0.146–0.214) than those prepared using the conventional bulk method. The Z-potential is a valuable parameter for predicting colloidal dispersions’ storage stability, as reported by Thode et al. [71]. When the zeta potential exhibits high negative values, it indicates the presence of electrostatic repulsion between particles, which prevents aggregation and stabilizes the nanoparticulate dispersion [18,45,72]. Moreover, negatively charged NPs have low toxicity to cells [73]. From the SEM images, it is clear that the prepared NPs had smooth surfaces. The sizes of blank and dye-loaded NPs formulated with both methods were within a range of 106–193 nm. When administered intravenously, NPs should be small enough (100–300 nm) to passively cross the tumor endothelial barrier and remain in the tumor bed for a prolonged period. This is due to reduced lymphatic drainage, which is also known as the enhanced permeability and retention (EPR) effect [16,74,75].

The release of fluorescein from both DNPs and MNPs followed a two-step pattern, with an initial rapid burst release observed during the first 4 h followed by a slower, sustained release over 24 h. Implementing a controlled and sustained release system is highly desirable for effectively maintaining optimal therapeutic drug levels over an extended period [76,77]. MNPs, which are smaller in size and have higher *EE_%_* and *DL_%_* than DNPs, may be better suited for applications requiring faster drug release rates. Further research in this area has the potential to lead to the development of more effective drug delivery systems with greater therapeutic efficacy.

The cytotoxicity assay revealed that although the cell viability gradually declined as the concentration of NPs increased from 0.01 to 10 mg/mL, both tested cell lines tolerated NPs well. We found that 120 nm-sized fluorescein-loaded NPs successfully entered the L929 and MG63 cells only after 1 h and accumulated in the cytosol, and it is plausible that some of the dye released may have diffused into the cell nuclei. In time, as in the fluorescent dye release assay, the dye was released and gathered in cells for 24 h. The next step should include drug encapsulation into PCL NPs and determining encapsulation efficiency. The further investigation of this method will allow the formulation of loaded NPs that could accumulate at the tumor sites via the EPR effect [74,75]. We believe that polymer-based microfluidic NP production will provide substantial opportunities for future clinical applications of size-controlled nanomedicines. A proposed extension of this work is to introduce other functional additives to tailor the properties of the nanoparticles for specific applications and to evaluate the effect of these substances on the NP precipitation process. The geometry of the mixing device can be easily modified to achieve better mixing and reduce the sizes and polydispersity of the nanoparticles obtained. The construction of the mixer device is simple, and it can be manufactured using a consumer-grade 3D printer and open-source software. The aspect ratio of the channels, defined as the width-to-height ratio, can influence the flow dynamics and NP formulation. A higher aspect ratio can promote better mixing while a lower aspect ratio can favor laminar flow conditions and enable easier control of NP size. The design of microfluidic channels can also incorporate various features such as bends, converging or diverging sections, and junctions to enhance mixing and achieve desired nanoparticle characteristics. It is worth noting that these dimensions are not fixed and can be tailored based on the specific requirements of the polymeric NP formulation process and the target application. Factors such as the viscosity of the polymer solution, the desired nanoparticle size range, and the intended drug loading or release characteristics can influence the selection of the microfluidic channel dimensions. The current study is the basis for further experiments addressing more specific applications of nanoparticles and improving mixing devices.

## Data Availability

The data presented in this study are available on request from the corresponding author.

## Supplementary Materials

The following supporting information can be downloaded at: https://www.mdpi.com/article/10.3390/polym15224375/s1, Table S1. Mean diameter and PDI of blank NPs. Size and polydispersity index (PDI) of PCL NPs measured by dynamic light scattering (DLS). Table S2. Comparison of dye-loaded NP characteristics formulated with analyzed methods. Size and polydispersity index (PDI) of PCL NPs with dye measured by dynamic light scattering (DLS). Figure S1. FTIR spectrum of dye-loaded NPs formulated with different amounts of PCL.
