# Supplementary material for: Microfluidic-Assisted Formulation of ε-Polycaprolactone Nanoparticles and Evaluation of Their Properties and In Vitro Cell Uptake
Source: Polymers (Basel). 2023 Nov 10;15(22):4375. doi: 10.3390/polym15224375 (PMC10674307; doi:10.3390/polym15224375)
Supplement: Supplementary file 1 [file polymers-15-04375-s001.zip › polymers-2601314-supplementary.pdf]

Supporting information for "Microfluidic-Assisted Formulation of ε-Polycaprolactone Nanoparticles and Evaluation of Their Properties and In Vitro and Cell Uptake"

Ewa Rybak [1,*], Piotr Kowalczyk [1], Sylwia Czarnocka-Śniadała [2], Michał Wojasiński [1], Jakub Trzciński [1,3,†] and Tomasz Ciach [1,2,†]

[1] Faculty of Chemical and Process Engineering, Warsaw University of Technology, Waryńskiego 1, 00-645 Warsaw, Poland; piotr.kowalczyk.dokt@pw.edu.pl (P.K.); michal.wojasinski@pw.edu.pl (M.W.); jakub.trzcinski@pw.edu.pl (J.T.); tomasz.ciach@pw.edu.pl (T.C.)

[2] Nanosanguis S.A., Rakowiecka 36, 02-532 Warsaw, Poland; s.czarnocka@nanogroup.eu

[3] Centre for Advanced Materials and Technologies CEZAMAT, Warsaw University of Technology, Poleczki 19, 02-822 Warsaw, Poland

* Correspondence: ewa.rybak.dokt@pw.edu.pl

† These authors contributed equally to this work.

Table S1. Mean diameter and PDI of blank NPs. Size and polydispersity index (PDI) of PCL NPs measured by dynamic light scattering (DLS).

	Dropwise		Microfluidic device	
PCL concentration [%]	Mean diameter ± SD [nm]	PDI [-]	Mean diameter ± SD [nm]	PDI [-]
0.1	122 ± 2	0.183 ± 0.010	122 ± 1	0147 ± 0.010
0.5	150 ± 4	0.090 ± 0.008	140 ± 2	0.150 ± 0.007
1.0	189 ± 2	0.146 ± 0.016	121 ± 4	0.140 ± 0.008
2.0	171 ± 3	0.187 ± 0.011	184 ± 1	0.060 ± 0.006
5.0	139 ± 2	0.252 ± 0.010	188 ± 3	0.123 ± 0.020

Table S2. Comparison of dye-loaded NP characteristics formulated with analyzed methods. Size and polydispersity index (PDI) of PCL NPs with dye measured by dynamic light scattering (DLS).

	Dropwise		Microfluidic device	
PCL concentration [%]	Mean diameter ± SD [nm]	PDI [-]	Mean diameter ± SD [nm]	PDI [-]
0.1	159 ± 3	0.424 ± 0.069	167 ± 5	0.201 ± 0.003
0.5	136 ± 5	0.585 ± 0.008	130 ± 2	0.199 ± 0.004
1.0	151 ± 0	0.653 ± 0.002	127 ± 3	0.180 ± 0.009
2.0	185 ± 4	0.154 ± 0.006	141 ± 5	0.146 ± 0.013
5.0	106 ± 2	0.609 ± 0.004	193 ± 2	0.214 ± 0.007

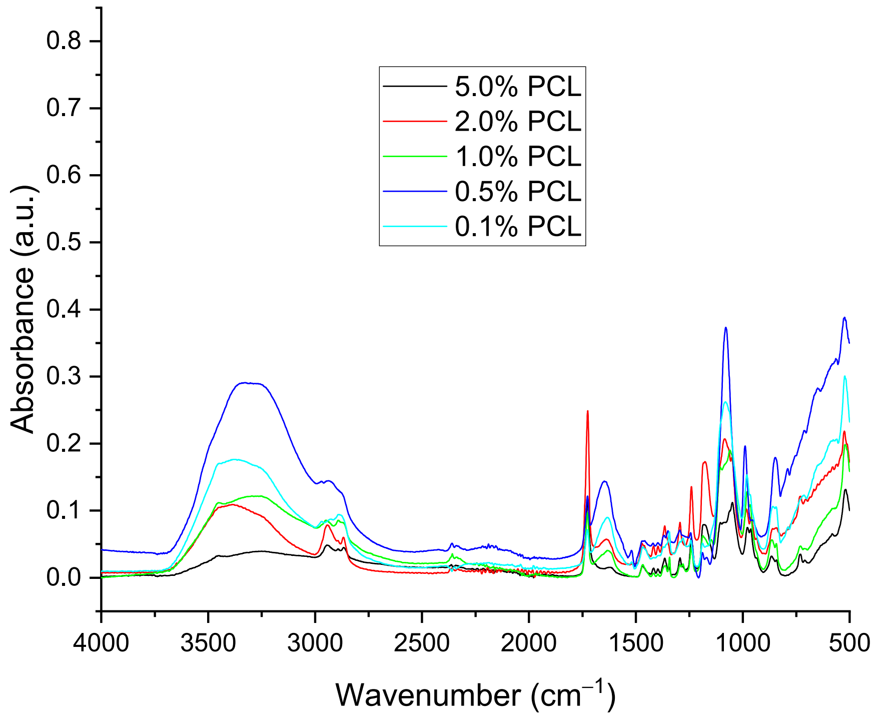

Figure S1. FTIR spectrum of dye-loaded NPs formulated with different amounts of PCL.
